# Six Year Refractive Change among White Children and Young Adults: Evidence for Significant Increase in Myopia among White UK Children

**DOI:** 10.1371/journal.pone.0146332

**Published:** 2016-01-19

**Authors:** Sara J. McCullough, Lisa O’Donoghue, Kathryn J. Saunders

**Affiliations:** Biomedical Sciences Research Institute, School of Biomedical Sciences, University of Ulster, Cromore Road, Coleraine, N. Ireland, United Kingdom; Sun Yat-sen University, CHINA

## Abstract

**Objective:**

To determine six-year spherical refractive error change among white children and young adults in the UK and evaluate differences in refractive profiles between contemporary Australian children and historical UK data.

**Design:**

Population-based prospective study.

**Participants:**

The Northern Ireland Childhood Errors of Refraction (NICER) study Phase 1 examined 1068 children in two cohorts aged 6–7 years and 12–13 years. Prospective data for six-year follow-up (Phase 3) are available for 212 12–13 year olds and 226 18–20 year olds in each cohort respectively.

**Methods:**

Cycloplegic refractive error was determined using binocular open-field autorefraction (Shin-Nippon NVision-K 5001, cyclopentolate 1%). Participants were defined by spherical equivalent refraction (SER) as myopic SER ≤-0.50D, emmetropic -0.50D<SER<+2.00 or hyperopic SER≥+2.00D.

**Main Outcome Measures:**

Proportion and incidence of myopia.

**Results:**

The proportion of myopes significantly increased between 6–7 years (1.9%) and 12–13 years (14.6%) (*p*<0.001) but not between 12–13 and 18–20 years (16.4% to 18.6%, *p* = 0.51). The estimated annual incidence of myopia was 2.2% and 0.7% for the younger and older cohorts respectively. There were significantly more myopic children in the UK at age 12–13 years in the NICER study (16.4%) than reported in Australia (4.4%) (*p*<0.001). However by 17 years the proportion of myopia neared equivalence in the two populations (NICER 18.6%, Australia 17.7%, *p* = 0.75). The proportion of myopic children aged 12–13 years in the present study (2006–2008) was 16.4%, significantly greater than that reported for children aged 10–16 years in the 1960’s (7.2%, *p* = 0.01). The proportion of hyperopes in the younger NICER cohort decreased significantly over the six year period (from 21.7% to 14.2%, *p* = 0.04). Hyperopes with SER ≥+3.50D in both NICER age cohorts demonstrated persistent hyperopia.

**Conclusions:**

The incidence and proportion of myopia are relatively low in this contemporary white UK population in comparison to other worldwide studies. The proportion of myopes in the UK has more than doubled over the last 50 years in children aged between 10–16 years and children are becoming myopic at a younger age. Differences between the proportion of myopes in the UK and in Australia apparent at 12–13 years were eliminated by 17 years of age.

## Introduction

There is a growing body of evidence suggesting that myopia is becoming more prevalent in childhood in many areas of the world, such as in Taiwan, [1] Singapore, [2] the United States [3] and Australia [4] while estimates of hyperopia prevalence remain relatively static.[4,5] Although myopia prevalence has been much studied, few prospective studies are available from which to derive estimates of incidence of myopia, the stability of hyperopia or to explore individual change in refractive error.[4,6–11] The present study reports the six-year change in refractive error status within a white, UK based population of children and young adults; exploring both the incidence of myopia and the stability of hyperopia in pre-teenage children, teenage children and young adults. Robust sampling and methodology similar to that used in other large-scale studies of refractive error [12,13] have been employed. Comparisons will be made with the recent report of six-year change in an Australian population of European Caucasian children [4] to determine geographical differences in an ethnically similar group and with historical data [14] to evaluate whether the refractive profile of UK school children and young adults has changed over the past 50 years.

## Materials and Methods

The Northern Ireland Childhood Errors of Refraction (NICER) Study is a longitudinal study of refractive error. The study methods have previously been described in detail [15] In brief, Phase 1 of the NICER study was a cross-sectional epidemiological study investigating the prevalence of refractive error in 6–7 and 12–13 year old children in Northern Ireland conducted between 2006 and 2008. Participants were chosen using stratified random sampling of schools from geographic areas characteristic of Northern Ireland to obtain a representative sample of schools and children from urban/rural and deprived/non-deprived areas. Data collection included cycloplegic autorefraction using the binocular open-field autorefractor (SRW-5000, Shin-Nippon, Tokyo, Japan). Cycloplegia was induced by one drop of 1.0% cyclopentolate hydrochloride, after corneal anaesthesia with one drop of 0.5% proxymetacaine hydrochloride. Autorefraction was performed at least 20 minutes after the instillation of drops. No less than five readings were taken from which the ‘representative value’ as determined by the instrument was used for further analysis. The representative value is widely used as an output value for this instrument and has recently been shown to be comparable to other methods of averaging refractive error.[16] Data collection occurred at the child’s school (6–7 year olds: primary school; 12–13 year olds: post-primary school) during the school day. After examination of the child, parents/guardians were asked to complete a questionnaire to determine the child’s birth history, family history and lifestyle. The study was approved by the University of Ulster’s Research Ethics committee and adhered to the tenets of the Declaration of Helsinki. Written informed consent was obtained from parents or guardians and verbal or written assent was obtained from participants on the day of the examination.

Within Phase 1 of the study, baseline data were collected from 399 6 to 7 year old children (younger cohort) and 669 12 to 13 year old children (older cohort). Phase 2 of the NICER study, collected follow-up data on the participants three years later, these data are presented elsewhere.[11] Phase 3 of the NICER study collected follow-up data on the same participants six years after their initial participation, 2012–2014. For the younger cohort, the majority of data collection at Phase 3 occurred at the child’s post-primary school (n = 200, 93%) and for some children where it was not possible to carry out testing at their post-primary school, data collection occurred at a University of Ulster campus (n = 15, 7%). For participants within the older cohort, data collection took place at one of the University of Ulster campuses or at a data collection site close to the participant’s home address (e.g. local church hall).

Data collection protocols were the same at all phases of the study. Cycloplegic autorefraction was measured using the latest version of the binocular open-field autorefractor at Phase 3 (NVision-K 5001, Shin-Nippon, Tokyo, Japan). This instrument has been shown to be accurate and repeatable over a wide range of refractive errors.[17,18]

### Refractive Classifications

The representative value was used to calculate spherical equivalent refraction (SER) using sphere + cylinder/2. There was a strong correlation between SER data from right and left eyes (Spearman’s rho, all *p*<0.001) therefore only data from right eyes are presented. SER was used to group participants into the following refractive classifications: a participant was classified as a myope if SER was -0.50 dioptre (D) or less; an emmetrope if SER was greater than -0.50D but less than +2.00D; and a hyperope if SER was +2.00D or greater. These classifications are similar to those used previously to define refractive error by the NICER study, [11,19] by the Refractive Error in School Children Study (RESC), [12,20] the Sydney Myopia Study (SMS)[13] and the Sydney Adolescent and Vascular Eye Study (SAVES).[4] Raw data from Sorsby *et al*.[14] were obtained and analysed using the same refractive criteria as the current study to aid comparison.

### Statistical Analysis

Data were analysed using Intercooled Stata 10.1 software (StataCorp LP, Texas, USA). Non-participants are defined as individuals who participated in Phase 1 of the study but did not participate in Phase 3. Differences between participants and non-participants for both cohorts were investigated using Mann-Whitney analysis (SER & socioeconomic rank), student t-tests (age) and chi-squared analysis (gender, refraction classification, spectacle wear at Phase 1, parental education & parental myopia). Socio-economic rank was determined using a Geographical Information Systems (GIS) approach. Unit postcode address information and the Northern Ireland multiple deprivation measure were applied to assign an area-based rank measure of economic deprivation to each child. The measure, calculated at the small scale census Output Area (OA) level, is based on three weighted domains of deprivation: income (41.7%), employment (41.7%) and proximity to services (16.6%). Level of parental education and parental myopia were established through parent/guardian questionnaire based survey at Phase 1. For parental education, participants were dichotomised as having at least one parent with third level education (college or university degree) or neither parent had third level education and for parental myopia, participants were dichotomised as having at least one myopic parent or having no myopic parents.

Cross-sectional distribution of refractive errors are presented for Phase 1 and Phase 3. Cumulative incidence of myopia was calculated as the number of individuals who were classified as myopic at Phase 3 but were not classified as myopic at Phase 1. Cumulative reduction in hyperopia (≥+2.00DS) was also assessed and is reported as the number of individuals classified as hyperopic at Phase 1 but not classified as hyperopic by Phase 3. Annual incidence of myopia (or reduction of hyperopia) were calculated by dividing the cumulative incidence (or reduction) by the mean follow-up interval (years) for each cohort as a whole. Six-year cumulative change in SER was calculated as “measurement at Phase 3”–“measurement at Phase 1”. Estimated annual change in SER was calculated by dividing the change from Phase 1 to Phase 3 by the time interval between examinations for each individual. The Shapiro-Wilk test was used to determine normality of the data. Mann-Whitney and Kruskal-Wallis tests or their parametric equivalents (Student t-test or analysis of variance) where appropriate, were used to analyse differences between cohorts and refractive error groups. Mixed effect logistic regression analyses and two sample tests of proportion were used to compare distribution differences within the NICER study and between the NICER study data and that of Sorsby *et al*.[14] and French *et al*. [4]. Chi-squared analyses were used for categorical and percentage comparisons. A *p* value of less than 0.05 was considered statistically significant.

## Results

### Participants

**Fig 1:**
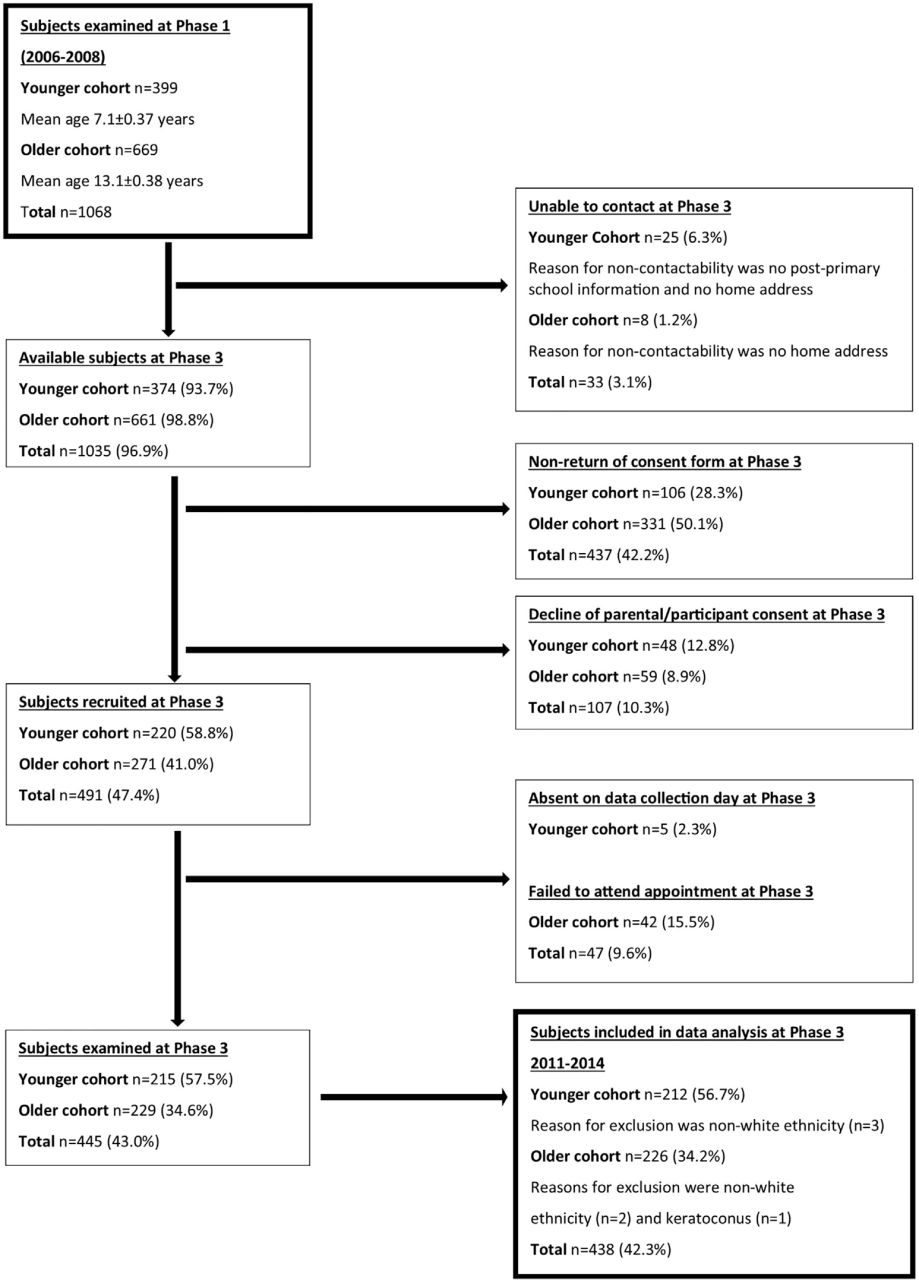
Flow diagram describing participant contactability, recruitment and exclusion from Phase 1 to Phase 3.

All participants who took part in Phase 1 were eligible to participate in Phase 3 irrespective of their participation in Phase 2. We were unable to contact a number of participants at Phase 3 in both the younger (n = 25, 6.0%) and older cohorts (n = 8, 1.2%). From Phase 1, overall participation in Phase 3 was 54% and 34% for the younger and older cohorts respectively. The majority of participants at Phase 3 were white (99%), reflective of the Northern Irish population, [21] therefore data are reported for white participants only. One participant in the older cohort was also excluded from data analysis due to emergent ocular pathology (keratoconus). Data are presented for 212 participants within the younger cohort (50% male) and for 226 participants within the older cohort (43% male). Fig 1 describes the number of participants contacted, recruited and examined at Phase 3.

#### Younger Cohort

The mean age of the younger cohort at Phase 3 was 13.1±0.4 years (range 12.4 to 13.9 years). There was no statistically significant difference between participants and non-participants at Phase 3 in terms of age (t = 1.39, *p* = 0.17), gender (Χ^2^ = 0.0014, *p* = 0.97), refractive error (SER [z = -0.18, *p* = 0.86] or refractive classification [Χ^2^ = 0.95, *p* = 0.81]), spectacle wearers vs non-spectacle wearers (Χ^2^ = 0.41, *p* = 0.52) and socio-economic indicators (socio-economic rank [z = -1.46, *p* = 0.14], and parental education [Χ^2^ = 0.05, *p* = 0.83]). Those who had at least one myopic parent were more likely to participate at Phase 3 compared to those with no myopic parents (Χ^2^ = 10.51, *p* = 0.001).

#### Older Cohort

The mean age of the older cohort at Phase 3 was 19.2±0.5 years (range 18.0 to 20.2 years). Females within the older cohort were statistically more likely to participate than males (Χ^2^ = 7.82, *p* = 0.005). There was no statistically significant difference in age (t = -1.90, *p* = 0.06), refractive error (SER [z = -0.40, *p* = 0.69] or refractive classification [Χ^2^ = 5.60, *p* = 0.13]), socio-economic indicators (socio-economic rank [z = -1.72, *p* = 0.09], parental education [Χ^2^ = 0.12, *p* = 0.73]) or parental myopia (Χ^2^ = 1.02, *p* = 0.31) between participants and non-participants at Phase 3. Spectacle wearers in the older cohort were statistically significantly more likely to participate at Phase 3 compared to non-spectacle wearers (Χ^2^ = 5.45, *p* = 0.02) however the range of spherical error of those who were spectacle wearers was wide (-7.00D to +9.00D).

### Follow-up Interval

The majority of follow-up examinations at Phase 3 occurred within 72 ±3 months of the initial participation in Phase 1 (younger 92.5%; older 73.9%). There was no statistically significant difference between cohorts with regard to the time interval between Phase 1 and 3 examinations (Mann-Whitney z = 0.71, *p* = 0.48) (younger median 73.2 months, IQR 70.3 to 73.9 months, range 68.8 to 76.7 months; older median 72.5 months, IQR 69.7 to 74.1 months, range 67.7 to 81.8 months).

### Incidence of Myopia and Reduction in Hyperopia between Phase 1 and Phase 3

**Fig 2:**
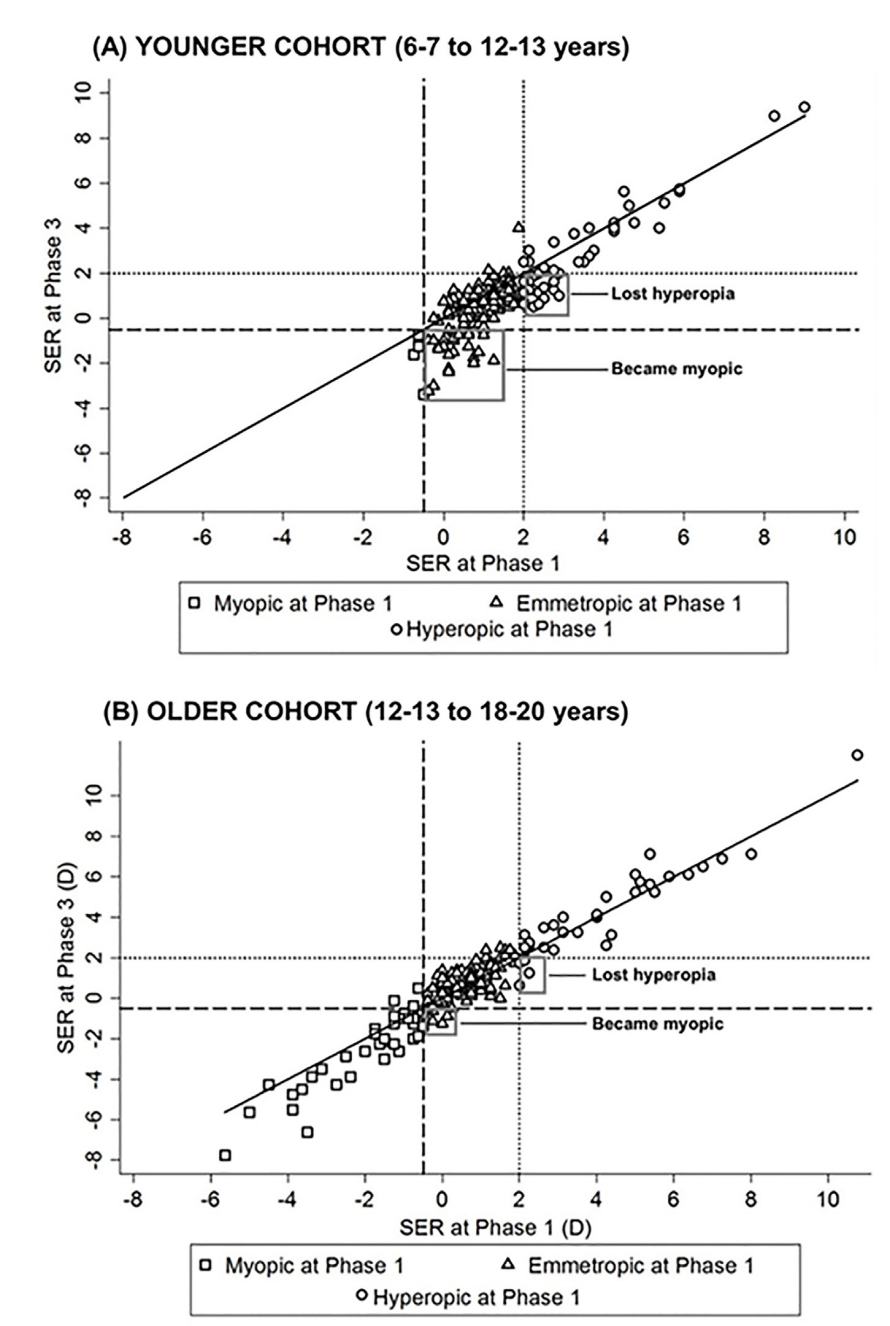
Scatterplots of SER at Phase 1 versus SER at Phase 3 for the younger and older cohorts respectively. Data are grouped into refractive classifications as illustrated in the key. The gray boxes indicate participants who became myopic (younger cohort n = 27; older cohort n = 8) or lost their hyperopia (younger cohort n = 20; older cohort n = 4) between Phase 1 and 3. The dashed lines represent the myopia cut-off point of -0.50D or less and the dotted line represents the hyperopia cut-off of +2.00D or greater. The solid black line represents unity- those falling below the line are showing a myopic change in SER.

Tables 1 and 2 describe the changing distribution of myopia and hyperopia for the younger and older cohorts. SER at Phase 1 and 3 are also plotted (Fig 2A & 2B) to show the number of new myopes by Phase 3 and the refractive classification in which they were originally grouped at Phase 1. The number of participants with reducing hyperopia are also highlighted.

**Table 1:** Proportion of myopes and incidence of myopia between Phase 1 and 3.

Cohort	Proportion of Myopes Phase 1 (%)	Proportion of Myopes Phase 3 (%)	*p*	Cumulative Incidence (%)	*p*	Estimated Annual Incidence (%)
**Younger**						
**All (n = 212)**	1.9 (n = 4)	14.6 (n = 31)	**<0.001**	13.0	-	2.2
**Males (n = 105)**	1.0 (n = 1)	14.3 (n = 15)	**0.006**	13.5	Reference	2.3
**Females (n = 107)**	2.8 (n = 3)	15.0 (n = 16)	**0.005**	12.5	0.63	2.1
**Older**						
**All (n = 226)**	16.4 (n = 37)	18.6 (n = 42)	0.51	4.2	-	0.7
**Males (n = 98)**	15.5 (n = 16)	16.5 (n = 17)	0.84	2.4	Reference	0.4
**Females (n = 128)**	17.1 (n = 21)	20.2 (n = 25)	0.49	5.6	0.38	0.9

Summary of the proportion of myopes at Phase 1 and 3 and the incidence of new myopes between Phase 1 and Phase 3 for the younger and older cohorts, stratified by gender. n = number of participants

**Table 2:** Proportion of hyperopes and decline of hyperopia between Phase 1 and 3.

Cohort	Proportion of Hyperopes Phase 1 (%)	Proportion of Hyperopes Phase 3 (%)	*p*	Cumulative Reduction (%)	*p*	Estimated Annual Reduction(%)
**Younger**						
**All (n = 212)**	21.7 (n = 46)	14.2 (n = 30)	**0.04**	43.5	-	7.3
**Males (n = 105)**	21.0 (n = 22)	13.3 (n = 14)	0.14	50.0	Reference	8.3
**Females (n = 107)**	22.4 (n = 24)	15.0 (n = 16)	0.15	37.5	0.97	6.3
**Older**						
**All (n = 226)**	15.0 (n = 34)	17.7 (n = 40)	0.44	11.8	-	2.0
**Males (n = 98)**	17.4 (n = 17)	19.4 (n = 19)	0.71	5.9	Reference	1.0
**Females (n = 128)**	13.3 (n = 17)	16.4 (n = 21)	0.48	17.6	0.39	2.9

Summary of the proportion of hyperopes at Phase 1 and 3 and the decline of hyperopia between Phase 1 and Phase 3 for the younger and older cohorts, stratified by gender. n = number of participants.

For those participants within the younger cohort classified as myopic at Phase 3 the cumulative median change in SER was -1.38D (IQR -0.63 to -2.75D) over the six-year period. Estimated annual median change for this group of participants was -0.23D (IQR -0.11 to -0.45D) with the largest estimated annual change for one individual of -0.51D. For the older cohort, those classified as myopic at Phase 3 showed a cumulative median change of -0.63D (IQR -0.13 to -1.00D) over the six-year period. Estimated annual median change for these participants was -0.10D (IQR -0.02 to -0.17D) with the largest estimated annual change of -0.51D. There was no statistically significant difference between genders for the annual rate of change in SER for those classified as myopic by Phase 3 for either the younger (Mann-Whitney, z = 0.41, *p* = 0.68) or older cohorts (Mann-Whitney, z = -1.26, *p* = 0.21).

There was no statistically significant difference between the cross-sectional proportion of participants aged 12–13 years classified as myopic at baseline (2006–2008, 16.4%) and those classified as myopic at Phase 3 (2012–2014, 14.6%) (Two-sample test of proportion z = -0.51, *p* = 0.62). Participants in both cohorts who were classified as myopic at 12–13 years of age had similar levels of myopia (median = -1.25DS, IQR -0.81 to -1.69DS in 2006–2008; median = -1.25D, IQR -0.88 to -2.38DS in 2012–2014) (Mann-Whitney, z = -0.85, *p* = 0.40).

There was a significant decline in the proportion of hyperopes in the younger cohort between age 6–7 years (21.7%) and 12–13 years (14.2%) (z = -2.05, *p* = 0.04). Children who were hyperopic at 6–7 years and remained hyperopic at 12–13 years had a significantly more positive SER at 6–7 years (median +3.69DS) compared to those who lost their hyperopia (median +2.19DS at 6–7 years) (Mann-Whitney, z = 4.62, *p*<0.001). The proportion of hyperopes within the older cohort remained relatively stable between 12–13 years (15.0%) and 18–20 years (17.7%) and there were few participants who lost their hyperopia over the six-year period (n = 4). Similar to the younger cohort, participants who were classed as hyperopic aged 12–13 years and remained hyperopic at age 18–20 years had a significantly greater SER at 12–13 years of age (median +4.13DS) compared to those who lost their hyperopia (median +2.07DS at 12–13 years) (Mann-Whitney, z = 2.84, *p* = 0.005). Hyperopic participants showed on average an annual change in SER of -0.09DS (IQR -0.17 to -0.02DS) and +0.02DS (IQR -0.04 to 0.11DS) for the younger and older cohorts respectively.

### Proportion, Incidence and Progression of Myopia among the NICER Study participants compared with a contemporary Sydney population and a historical UK based population

#### Proportion of Myopia

**Fig 3:**
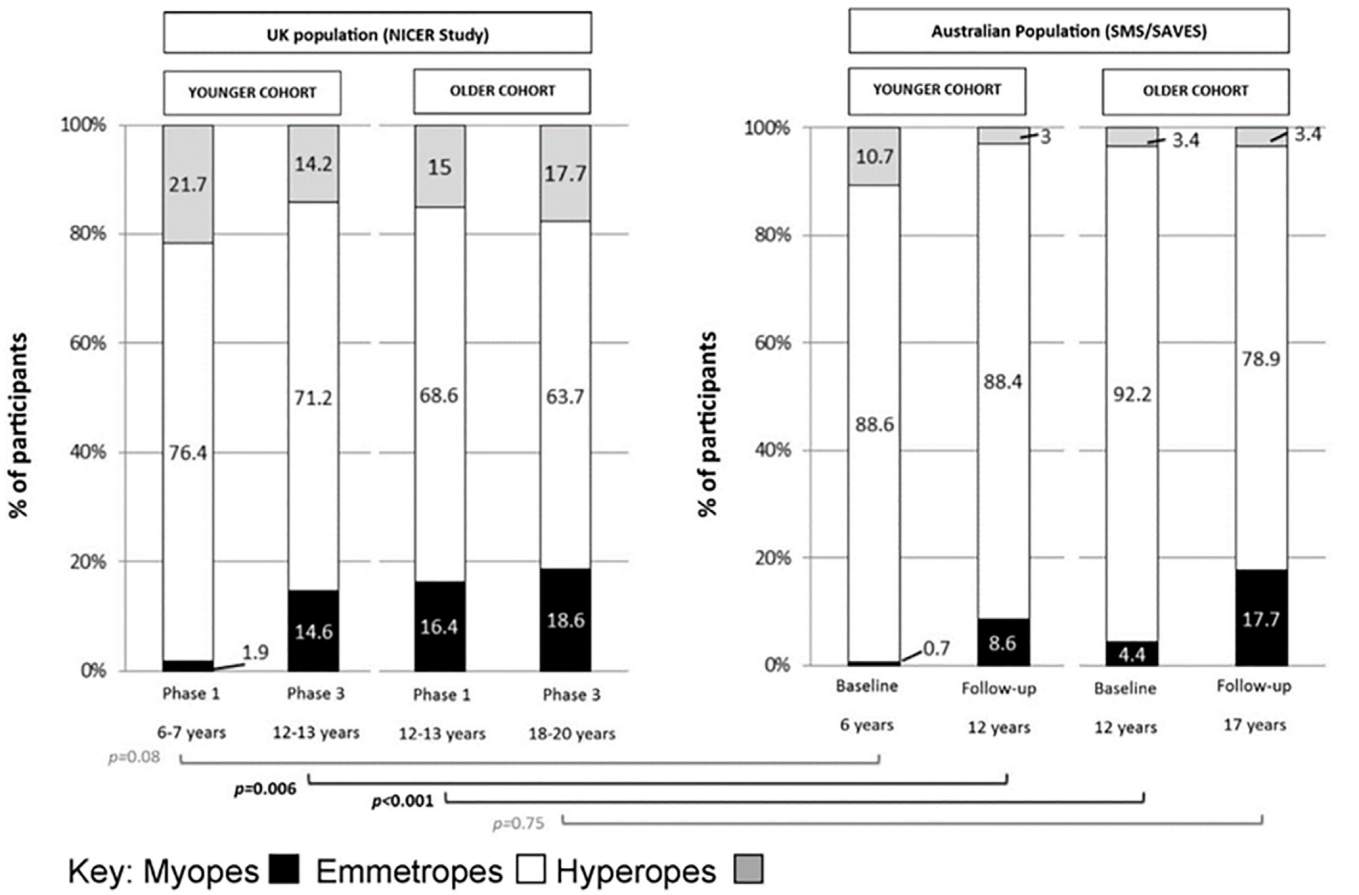
Comparison of refractive error prevalence in the UK (NICER Study) to Australia (SMS/SAVES) at baseline and 5–6 year follow-up. The brackets show the statistical comparisons of the proportions of myopes between the two studies; black indicates statistically significant difference, gray indicates no statistically significant difference (Two sample test of proportion).

Data from the current study are compared with data from the recent report from French *et al*.[4] (Sydney Myopia Study [SMS] and Sydney Adolescent Vascular and Eye Study [SAVES]) who present the prevalence, incidence and progression of myopia in two similar age cohorts of Australian European Caucasian children (aged 6 years and 12 years at baseline) and longitudinal follow-up after 5–6 years during a similar time period as the present study (2004 to 2011). These data are presented in Fig 3.

**Fig 4:**
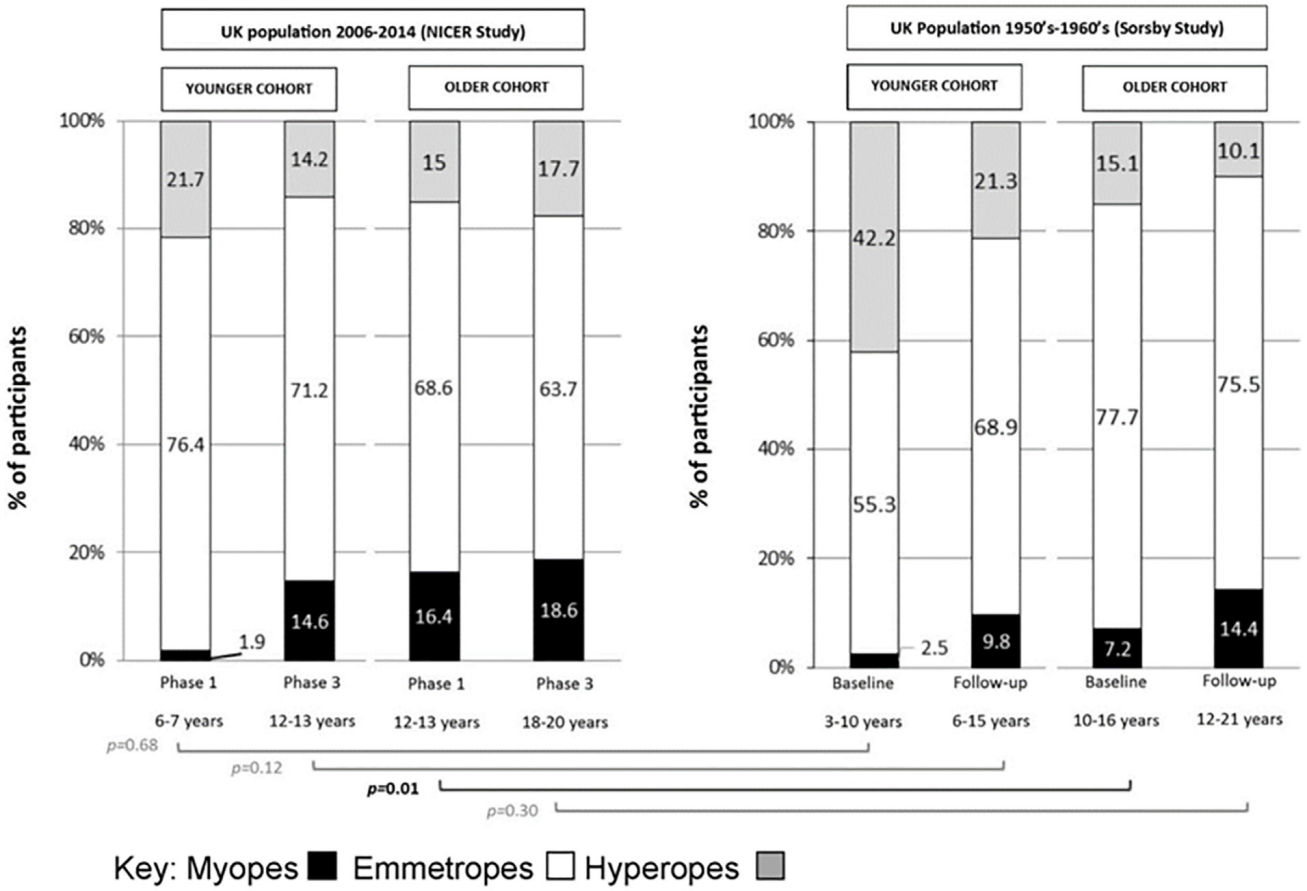
Comparison of UK refractive error distribution between 1950’s-1960’s (Sorsby study) and 2006–2014 (NICER study) at baseline and follow-up. The brackets indicate the statistical comparisons of the proportions of myopes between the two studies; black indicates statistically significant difference, gray indicates no statistically significant difference (Two sample test of proportion).

Data from the current study are also compared with historical UK data from Sorsby *et al*.[14] (Fig 4). Sorsby *et al*. present data for two age groups; children who were between three and ten years of age at baseline (younger cohort) and children who were ten to 15 years of age at baseline (older cohort). Longitudinal data are also presented with a mean follow-up examination occurring at 3.1±0.9 years (range 2.2 to 5.3 years) and 3.7±0.9 years (range 2.0 to 5.3 years) for their younger and older cohorts respectively.

**Fig 5:**
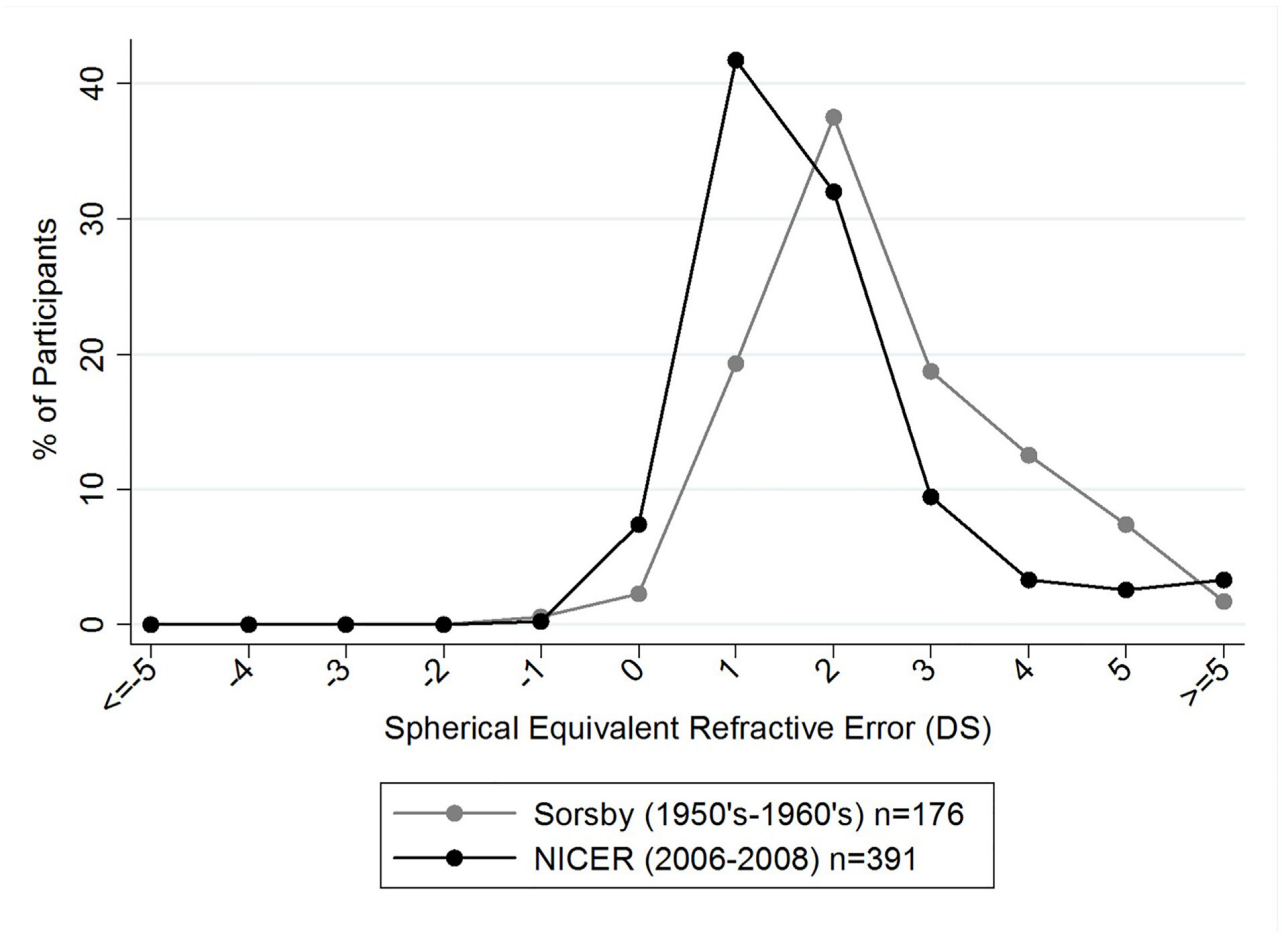
Distribution of spherical equivalent refractive errors in 6–7 year old children within the NICER study Phase 1 (2006–2008) and 6–7 year old children from Sorsby *et al*.[14]. Data points represent a one dioptre interval (for example, the % of participants represented at point 0 on the x-axis have an SER of less than or equal to 0DS but greater than -1 DS. Data points at the extremes of the x-axis represent participants with SER of greater than or equal to +5DS or less than or equal to -5DS.

In contrast to contemporary literature, and to the data presented in Fig 4, Sorsby *et al*.[14] applied an SER of less than zero dioptres to define myopia in his published report. Using this criterion and applying it to the NICER study data, 23% of children aged 12–13 years in the NICER study were classified as myopic at Phase1 (2006–2008) compared to 10% reported by Sorsby *et al*. in the 1960’s for their older cohort of children aged between 10–16 years. These data and those in Fig 5 indicate a two-fold increase in the proportion of myopia in the UK over the last five decades in children aged between 10 and 16 years (Two sample test of proportion z = 3.12, *p* = 0.002).

Fig 5 presents the distribution of SER for 6–7 year old children within Phase 1 of the NICER study (2006–2008) and from Sorsby’s report.[14] The median SER for Sorsby’s 6–7 year olds in the 1950’s-1960’s was +1.80DS (IQR +1.30 to +2.70DS) which is significantly more hyperopic than the median SER (+1.13DS, IQR +0.63 to +1.75DS) for the 6–7 year olds in the NICER study in 2006–2008 (Mann-Whitney z = 7.15, *p*<0.001).

Table 3 describes the mean annual change in SER for those classified as myopic at baseline and the estimated annual incidence of myopia for the NICER and Sydney data. The Sydney study [4] reported no statistically significant difference in the annual progression rate between children of European Caucasian or East Asian ethnicity when myopia was present at baseline, therefore change in SER data for all ethnicities in the Sydney study are used for comparison. Isolated European Caucasian data are not available for the Sydney data for this metric.

**Table 3:** Comparison of the incidence and progression of myopia between the NICER and Sydney studies for the younger and older cohorts.

		YOUNGER COHORT	OLDER COHORT
**Current Study NICER**	**Age range**	**6–7 to 12–13 years**	**12–13 to 18–20 years**
	**Estimated Annual Incidence of myopia**	2.2%*	0.7%*
	**Mean Annual Change in SER for participants classed as myopic at Phase 1 (D) (Range)**	n = 4; -0.18; (-0.47 to -0.02)	n = 37; -0.09; (-0.51 to +0.19)
**Sydney, Australia French *et al*.[4]**	**Age Range**	**6–7 to 12–13 years**	**12–13 to 17 years**
	**Estimated Annual Incidence of myopia**	1.3%*	2.9%* †
	**Mean Annual Change in SER for participants classed as myopic at Phase 1 (D)**	n = 11; -0.41; (range not available)	n = 128; -0.31; (range not available)

n = number of participants.

* = statistically significant difference in annual incidence between younger and older cohorts within the same study (Χ^2^, *p*<0.05).

^†^ = statistically significant difference in annual incidence between the NICER study and the Sydney data (within the same age cohort) (Χ^2^, *p*<0.05).

## Discussion

The present study reports the six-year change in refractive error status including the incidence of myopia and reduction of hyperopia, within a white, UK based population of children and young adults. In the present study, the proportion of participants defined as myopic is similar to previous reports of European Caucasian children of similar age.[4,10,22,23] However it is relatively low in comparison to reports among children of Asian background whether living in Asia or elsewhere.[1,4,6,8,20,23] French *et al*.[4] report a significant increase in the prevalence of myopia in Australian Caucasian children between 2004 and 2011 and infer that the prevalence of myopia is increasing in Australia, similar to reports in many other parts of the world.[1–3] The results from the present study demonstrate a two-fold increase over 50 years in the proportion of teenagers who are myopic in the UK, but no significant short-term increases in myopia prevalence during the past decade in our population.

### Proportion and Onset of Myopia

There was a statistically significant increase in the number of myopes between 6–7 and 12–13 years. The estimated annual incidence in myopia within this younger cohort (2.2%) did not differ significantly from that found in European Caucasian children in Sydney (1.3%) and is comparable with annual incidences reported in the ethnically diverse populations described in the full Sydney data set (2.2%) (French *et al*.[4]-including all ethnicities) and in the Collaborative Longitudinal Evaluation of Ethnicity in Refractive Error (CLEERE) study. The latter reports an annual incidence of myopia of 2.8% for children in the United States aged six years at baseline who were reviewed annually for seven years.[24] In general, children living within the UK, US and Australia appear to have similar low annual incidences of myopia, regardless of ethnicity, compared to the high annual incidences reported for children living in East Asia. Saw *et al*.[8] report an annual incidence of myopia of 15.9% among Singaporean children aged seven years at baseline (reviewed after three years) and Fan *et al*.[6] report on average a 14.4% annual incidence of myopia among children from Hong Kong (aged between 7–11 years at baseline, reviewed after one year). Prospective data from ethnically diverse groups living in the UK would be beneficial to identify how ethnicity and environment interact in the UK.

Gender did not significantly impact on myopia incidence in the NICER study in either age cohort. Although the proportion of myopes who were females was higher than males in both cohorts, this did not reach statistical significance. Zhao *et al*.[20] in the Refractive Error of School-Children (RESC) study in the Shunyi District, China, report an annual incidence of myopia of 2.2% among children who were five years old at baseline (followed up after 28.5 months), similar to our younger cohort. However among children who were 12 years at baseline in Zhao *et al*.’s study, the annual incidence of myopia had risen to 10.7% for males and 16.7% for females in stark contrast to the present study. French *et al*.[4] also report significantly greater myopia incidence in females in their older group of children (aged 12–13 years at baseline) but no significant difference between genders for their younger cohort (aged 6–7 years at baseline). Saw *et al*.[8] also report a higher annual incidence of myopia in female Singaporean children (15.2%) (aged 7–9 years at initial visit, reviewed after three years) compared to males (13.2%) however similar to the NICER study, this difference did not reach statistical significance.

Within the present study there was no statistically significant increase in the proportion of participants classed as myopic between 12–13 years and 18–20 years of age. The cumulative incidence of myopia for this cohort was 4.2% with an estimated annual incidence of 0.7%. By contrast, the younger cohort demonstrated a greater cumulative incidence of myopia in the six-year sampling period (13% vs 4.2%) revealing that children were three times more likely to become myopic between 6–7 and 12–13 years than between 12–13 and 18–20 years in our population. Compared to Sorsby *et al*.[14] (Fig 5) our results show that children are becoming myopic at a younger age in present day UK compared to 50 years ago. Children within the NICER study also demonstrated a significantly less hyperopic SER at 6–7 years of age compared to those of corresponding age within Sorsby’s study. Williams *et al*.[25] have recently reported a similar trend of increasing myopia prevalence in adults in Northern and Western Europe where the prevalence of myopia was almost twice as high in young adults aged 25–29 years (47.2%) compared to those of middle age (27.5%, aged between 50–59 years). They also report a significantly higher prevalence of age-standardised myopia among adults born between 1940–1979 (23.5%) compared to those born between 1910–1939 (17.8%).

Cumulative incidence of myopia decreased with increasing age from the younger to the older NICER cohort. This contrasts strongly with data in a sample of Chinese children (aged 5–15 years at baseline, reviewed after 28.5 months) for which Zhao *et al*.[20] report a 27% increase in cumulative risk of myopia with each additional year of age. French *et al*.[4] also report that the incidence of myopia increased with increasing age among their Australian children, describing the annual incidence of myopia in their older cohort (aged 12 years at baseline) as 2.9% compared with 1.3% in their younger cohort (aged six years at baseline) with an annual incidence of 1.3%. Inspection of Fig 3 suggests that although the prevalence of myopia among younger Australian children is lower than that found in our UK population, by age 17 years the proportion of myopes in the two studies nears equivalence. It is also seen in Fig 3 and reported by French *et al*.[4] that cross-sectional data indicate a shift towards earlier onset myopia in Australia in a relatively short space of time; the prevalence of myopia in 12–13 year old children tested in 2004–2005 is reported as 4.4% compared with 8.6% in 2009–2011. This shift has been attributed to a change in lifestyle amongst younger Australian children; including increased use of computers and hand-held technology and less time spent playing outside. Spending time outdoors has been postulated to protect against the onset and progression of myopia [26–29] and French et al.[28] suggests that while young children may have traditionally been protected by the sunny climate and outdoor lifestyle prevalent in Australia, modern lifestyle pressures mean that time outdoors is sacrificed to the growing demands of study and the attraction of computer games and tablets, and hence susceptibility to myopia is intensified. The data from the present study do not support a rapid change in the timing of myopia onset in the UK as reported in Australia, but do reveal that myopia occurs at a younger age in the UK in the 21^st^ century than reported in the 1960’s. This, in addition to the evidence of a twofold increase in the number of myopes, is likely to reflect the significant changes in lifestyle and environment that have occurred over the last 50 years in the UK (e.g. time spent indoors, use of electronic devices, change in diet, obesity, onset of puberty, sedentary lifestyles).

### Rate of Change

The annual rate of change of SER for those classified as myopic at Phase 1 was greater at 6–7 years than at 12–13 years similar to trends reported in other white [4,14] and Asian childhood populations.[8] The annual rate of change for those classified as myopic at baseline is much greater in the Sydney study (Table 3) across both the younger and older cohorts than for the NICER study participants. In the younger cohort of the NICER study the annual rate of change of SER from 6–7 to 12–13 years for those who were classified as myopic by Phase 3 (n = 31) also shows myopic progression occurring at a slower rate than their Australian contemporaries. Pärssinen and Lyyra [30] report an average annual change of -0.55D among myopic Finnish children who were approximately 10 years old at baseline and were reviewed every year for three years, and report that females progressed significantly faster than males. This annual rate of progression is over twice that of the younger cohort of the present study and in contrast to Pärssinen and Lyyra we found no significant difference in the incidence or progression of myopia between genders. The average annual change in SER for participants who were classified as myopic at Phase 3 was -0.23D and -0.10D for the younger and older cohorts respectively. Considering a clinically significant change in SER to be -0.25D or more, our data suggest that to ensure children have appropriate, up-to-date refractive correction the following guidance could be applied: those children aged 6–7 years who are myopic or at risk of developing myopia (e.g. those with a positive family history, those who are emmetropic or have low levels or hyperopia (<+0.75D) in early childhood, those with sedentary lifestyles, those spending less than three hours outdoors per day-[31–36]) should be advised to have an annual eye examination. At 12–13 years, myopic children or for those at risk of developing myopia (as above for 6–7 years and in addition those in academically selected schooling [35]) it may be appropriate to extend this routine sight test interval to two years unless symptoms indicate a need for more rapid intervention. This guidance relates only to monocular spherical equivalent refractive error and does not consider the dynamics of other visually important refractive features such as astigmatism and anisometropia. Clinicians should be aware that these data apply to a UK based population where myopia progression appears slower than that of other populations.

### Change in Hyperopia

The proportion of participants classified as hyperopic within the present study was high for both cohorts in comparison to the Sydney population [4] but comparable to that recorded under cycloplegia by Logan *et al*.[23] for white UK children of the same age. Czepita *et al*.[22] report a higher prevalence of hyperopia (30.8%) in Polish children aged 10–14 years living in rural areas compared to urban areas (7.1%) in Poland. Northern Ireland has a relatively rural population (population density of 250–1000 per km^2^)[37] which is similar to that of rural Poland (population density of <1000 per km^2^)[38] and in contrast to the urban population of Sydney (7000-8000/km^2^).[39] However, Logan *et al*.’s data represent children schooled in a large UK city and present a relatively similar situation to that found in Northern Ireland. Further work is needed to fully understand why hyperopia is more common in some populations than in others.

The present study reports a significant annual reduction in hyperopia of 7.3% within the younger cohort as they progress from 6–7 to 12–13 years of age. There are few studies which have prospectively investigated change in hyperopia, however French *et al*.[4] report a greater annual rate of reduction in hyperopia over a 5–6 year time period in both their cohorts of Australian children living in Sydney (aged six and 12 years at baseline) compared to the present study; with an annual reduction of hyperopia of 12.3% and 10.2% for the younger and older cohorts respectively. French *et al*.’s data are from children of mixed ethnicity (22% East Asian) which may explain the greater myopic shift in refractive error compared to the present study. Zhao *et al*.[20] also report a large annual reduction of hyperopia of 17.6% among children within the Shunyi District of China (aged 5–15 years at baseline). Baseline prevalence of hyperopia within Zhao’s Chinese population was also much lower (3%) than that found in the NICER study.

For both cohorts within the present study, those whose hyperopia was greater than +3.50DS showed a relatively stable SER over the six-year period, in contrast to less hyperopic peers whose hyperopia tended to reduce towards emmetropia during the study. Prospective studies of infants’ and young children’s refractive development also demonstrate that hyperopia that fails to resolve through emmetropisation within the first year or two of life is likely to be persistent.[40–42] Mutti *et al*.[43] states that infants with hyperopic errors of 4D or more are significantly less likely to emmetropise than infants with lower levels of hyperopia and that hyperopia of this level or greater is persistent. We have demonstrated that this persistent infantile hyperopia endures through later childhood and early adulthood. Several researchers have identified an association between retention of significant hyperopia in infancy (4D or more) and poor accommodative function [44–47]. When retained beyond infancy higher levels of hyperopia signal a resistance of the visual system to modification through visual feedback usually associated with emmetropisation. Kulp *et al*.[48] have suggested that the presence of heterotropia may reduce the rate of hyperopic decline and within our study, one third of those with persistent hyperopia demonstrated heterotropia. In contrast to studies exploring hyperopic decline in childhood in other geographic and ethnic populations [4,49], the current study reports a greater proportion of significant hyperopia throughout childhood and early adulthood and a much lower annual rate of refractive change in hyperopia. It appears that genetic or environmental variance influences the persistency of hyperopia. Our data would support Mezer *et al*.’s [49] findings that children with mild hyperopia may be able to cease spectacle wear in later childhood, however those with moderate to high hyperopia will need to retain their refractive correction.

### Strengths and Limitations

Study methods and refractive classifications used in the NICER study were similar to other large studies of refractive error in children for ease of comparison.[4,12,23] The epidemiological design of the initial NICER study did not give consideration to sample size for future prospective studies, therefore sample sizes at six year follow-up while favourable in comparison to the only other published UK based prospective study evaluating cycloplegic refractive data in childhood, [14] are modest in comparison to other published data.[4,20,31] While most participants in the present study were contactable at Phase 3 and participation rates are comparable to other longitudinal studies over similar time frames, [4,31] a significant number of participants were not re-examined particularly within the older cohort. Within the younger cohort, there were no differences between those who participated at Phase 3 and those lost to follow-up in age, gender, refractive error and socioeconomic factors. Children who had at least one myopic parent were more likely to participate at Phase 3 than those with no myopic parents which may have a genetic influence on the incidence of myopia within the younger cohort.

Within the older cohort, females were significantly more likely to participate than males. We also report a higher percentage of myopic females than males and a greater incidence of myopia in females to males within this cohort; however the differences were not statistically significant. These retention issues could potentially bias our data towards more myopia in the older cohort. Within the older cohort, spectacle wearers were more likely to participate at Phase 3 than non-spectacle wearers possibly due to a greater interest in their eye care, however spectacles wearers included both hyperopes and myopes.

The NICER study data have been compared with the data from Sorsby *et al*.[14] and although efforts have been made to compare refractive classifications and rate of change of myopia, there are limitations in these comparisons due to differences in the exact age of the cohorts compared, the informal sampling methodology, method of refraction and lack of uniformity in the follow-up intervals in Sorsby’s study. Data from 6–7 year old children from the Sorsby study have been directly compared with 6–7 year old children from the NICER study due to a large number of data points within the Sorsby study at this age; participant numbers in the Sorsby study were limited in other comparable age groups to the NICER study and have not been directly compared. Given that Sorsby’s work is widely cited and well known in the UK and beyond, it is useful to examine it in light of the contemporary findings of the NICER study in an attempt to explore how refractive error has altered over the 50 year period.

The data presented on change in refractive error within this study are supported by ocular biometric data that are presented in Supporting Information (S1, S2, S3 and S4 Tables).

## Conclusions

In comparison to other worldwide studies the proportion of children and young adults classified as myopic remains relatively low in this white, UK based population and the proportion of myopes is similar to other populations of European Caucasians of similar age. Differences between the proportion of myopes in the NICER study and a comparable study of Australian children of European Caucasian ethnicity apparent at 12–13 years were eliminated by 17 years of age. Our data suggest that the proportion of myopes has remained relatively stable in the UK over the short-term but has doubled in the last 50 years. Our results also suggest white children in the UK are becoming myopic at a younger age than previously demonstrated; children are more likely to develop myopia in the UK between 6–7 and 12–13 years than during teenage years. Hyperopic errors above +3.50D tend to be persistent and stable across the school years, but lower levels of hyperopia often decrease.

## Supporting Information

S1 Table Raw data set for the change in ocular biometrics (AL = axial length, corneal power & ACD = anterior chamber depth) and SER (spherical equivalent refraction) between Phase 1 and Phase 3 (6–7 years to 12–13 years) for participants classified as myopic within the younger cohort. Participants shown in bold were classified as myopic at both Phase 1 and Phase 3. Outlined below are the Spearman correlations between the change in SER and change in AL, corneal power and ACD. Change in SER vs Change in AL, Spearman’s Correlation, ρ = -0.7510, *p*<0.001. Change in SER vs Change in Corneal Power, Spearman’s Correlation, ρ = -0.036, *p* = 0.985. Change SER vs Change in ACD, Spearman’s Correlation, ρ = -0.347, *p* = 0.061.
(PDF)

S2 Table Raw data set for the change in ocular biometrics (AL = axial length, corneal power & ACD = anterior chamber depth) and SER (spherical equivalent refraction) between Phase 1 and Phase 3 (12–13 years to 18–20 years) for participants classified as myopic within the older cohort. Participants shown in bold were classified as myopic at both Phase 1 and Phase 3 and participants shown in italics were classified as myopic at Phase 1 but not at Phase 3. Outlined below are the Spearman correlations between the change in SER and change in AL, corneal power and ACD. Change in SER vs Change in AL, Spearman’s Correlation, ρ = -0.740, *p*<0.001. Change in SER vs Change in Corneal Power, Spearman’s Correlation, ρ = 0.045, *p* = 0.768. Change SER vs Change in ACD, Spearman’s Correlation, ρ = -0.302, *p* = 0.0047.
(PDF)

S3 Table Raw data set for the change in ocular biometrics (AL = axial length, corneal power & ACD = anterior chamber depth) and SER (spherical equivalent refraction) between Phase 1 and Phase 3 (6–7 years to 12–13 years) for participants classified as hyperopic at Phase 1 within the younger cohort. Outlined below are the Spearman correlations between the change in SER and change in AL, corneal power and ACD. Change in SER vs Change in AL, Spearman’s Correlation, ρ = -0.707, *p*<0.001. Change in SER vs Change in Corneal Power, Spearman’s Correlation, ρ = -0.114, *p* = 0.457. Change SER vs Change in ACD, Spearman’s Correlation, ρ = -0.520, *p*<0.001.
(PDF)

S4 Table Raw data set for the change in ocular biometrics (AL = axial length, corneal power & ACD = anterior chamber depth) and SER (spherical equivalent refraction) between Phase 1 and Phase 3 (12–13 years to 18–20 years) for participants classified as hyperopic at Phase 1 within the older cohort. Outlined below are the Spearman correlations between the change in SER and change in AL, corneal power and ACD. Change in SER vs Change in AL, Spearman’s Correlation, ρ = -0.594, *p*<0.001. Change in SER vs Change in Corneal Power, Spearman’s Correlation, ρ = 0.008, *p* = 0.964. Change SER vs Change in ACD, Spearman’s Correlation, ρ = -0.322, *p* = 0.063.
(PDF)
