# Supplementary material for: Six Year Refractive Change among White Children and Young Adults: Evidence for Significant Increase in Myopia among White UK Children
Source: PLoS One. 2016 Jan 19;11(1):e0146332. doi: 10.1371/journal.pone.0146332 (PMC4718680; doi:10.1371/journal.pone.0146332)
Supplement: S1 Table — Participants shown in bold were classified as myopic at both Phase 1 and Phase 3. Outlined below are the Spearman correlations between the change in SER and change in AL, corneal power and ACD. Change in SER vs Change in AL, Spearman’s Correlation, ρ = -0.7510, p<0.001. Change in SER vs Change in Corneal Power, Spearman’s Correlation, ρ = -0.036, p = 0.985. Change SER vs Change in ACD, Spearman’s Correlation, ρ = -0.347, p = 0.061. (PDF) [file pone.0146332.s001.pdf]

S1 Table

ID	Change in SER (D)	Change in AL (mm)	Change in Corneal Power (D)	Change in ACD (mm)
**BB14**	**-0.875**	**1.220**	**-0.186**	**0.100**
BB15	-1.000	1.120	-0.093	0.210
BB21	-1.875	1.390	0.030	0.230
BB23	-1.125	0.930	-0.089	0.220
BB24	-1.750	1.930	-0.251	0.510
BB28	-3.125	1.940	-0.304	0.210
BB37	-2.375	1.840	-0.075	0.320
BKeel12	-2.875	1.840	-0.023	0.200
**BKeel25**	**-0.625**	**1.090**	**0.080**	**0.160**
BKY01	-1.750	1.520	0.070	0.260
BKY04	-1.250	1.610	-0.214	0.320
BM12	-0.625	0.690	-0.052	0.170
CH10	-1.375	1.140	0.103	0.190
DH04	-2.375	.	.	.
DH13	-1.250	0.950	-0.019	0.130
DHCM1006	-1.750	0.912	-0.217	0.110
**DHCM2005**	**-0.125**	**0.940**	**-0.292**	**-1.120**
DR14	-1.250	1.110	0.065	0.170
GH23	-2.375	1.660	0.06	0.390
GH28	-1.250	1.140	-0.045	0.280
HB04	-2.500	1.920	0.205	0.290
HB19	-0.625	0.710	-0.098	0.150
LCEN007	-2.75	2.100	-0.174	0.240
**LCEN021**	**-2.875**	**1.870**	**-0.06**	**0.210**
LCEN036	-2.750	1.840	0.029	0.290
LIS08	-1.125	0.910	-0.303	0.070
LIS09	-0.750	1.090	0.355	0.980
LIS19	-1.125	0.890	0.064	0.220
LIS32	-0.875	1.180	-0.189	0.230
PR10	-1.500	0.620	0.102	0.160
SJ01	-2.500	1.410	-0.267	0.120
