# Supplementary material for: Six Year Refractive Change among White Children and Young Adults: Evidence for Significant Increase in Myopia among White UK Children
Source: PLoS One. 2016 Jan 19;11(1):e0146332. doi: 10.1371/journal.pone.0146332 (PMC4718680; doi:10.1371/journal.pone.0146332)
Supplement: S2 Table — Participants shown in bold were classified as myopic at both Phase 1 and Phase 3 and participants shown in italics were classified as myopic at Phase 1 but not at Phase 3. Outlined below are the Spearman correlations between the change in SER and change in AL, corneal power and ACD. Change in SER vs Change in AL, Spearman’s Correlation, ρ = -0.740, p<0.001. Change in SER vs Change in Corneal Power, Spearman’s Correlation, ρ = 0.045, p = 0.768. Change SER vs Change in ACD, Spearman’s Correlation, ρ = -0.302, p = 0.0047. (PDF) [file pone.0146332.s002.pdf]

## S2 Table

| id | Change in SER (DS) | Change in AL (mm) | Change in Corneal Power (D) | Change in ACD (mm) |
|---|---|---|---|---|
| **BA01** | **0.375** | **0.220** | **0.007** | **-0.080** |
| **BA06** | **-1.250** | **0.570** | **-0.229** | **0.020** |
| **BA07** | **-2.125** | **1.240** | **-0.198** | **-0.020** |
| **BA18** | **-0.500** | **0.410** | **0.028** | **0.120** |
| **BA24** | **-0.625** | **0.010** | **0.358** | **-0.150** |
| **BA29** | **-0.125** | **0.080** | **0.017** | **-0.010** |
| **BA56** | **-0.375** | **0.590** | **-0.064** | **0.010** |
| BA64 | -1.000 | 0.450 | -0.228 | 0.090 |
| *BC08* | *0.375* | *0.060* | *0.017* | *-0.020* |
| CHS07 | -0.875 | 0.950 | -0.419 | 0.200 |
| CHS10 | -1.250 | 0.580 | 0.140 | 0.070 |
| CHS18 | -0.125 | 0.540 | 0.108 | 0.150 |
| **CHS21** | **-0.500** | **0.380** | **-0.071** | **-0.100** |
| **CHS23** | **-0.875** | **0.250** | **0.066** | **-0.080** |
| **CHS24** | **-0.625** | **0.450** | **-0.017** | **-0.090** |
| *CHS31* | *1.125* | *0.080* | *-0.223* | *-0.040* |
| **CI08** | **-0.625** | **0.520** | **-0.076** | **0.060** |
| **CI18** | **-1.000** | **0.680** | **-0.028** | **0.140** |
| **CI35** | **-3.125** | **1.440** | **-0.289** | **0.050** |
| CI61 | -0.750 | 0.400 | -0.001 | 0.130 |
| **LC05** | **-0.625** | **0.760** | **-0.066** | **0.060** |
| **LC09** | **-1.500** | **0.800** | **0.175** | **0.160** |
| **LC17** | **-1.250** | **0.490** | **1.123** | **0.060** |
| **LC32** | **0.000** | **0.380** | **-0.031** | **-0.030** |
| **LC37** | **-0.875** | **0.500** | **0.118** | **0.010** |
| **LG01** | **0.000** | **0.160** | **0.073** | **0.040** |
| **LG09** | **0.250** | **0.290** | **0.478** | **0.010** |
| **LG17** | **-0.250** | **0.440** | **0.408** | **0.130** |
| **LG28** | **0.000** | **0.680** | **-0.098** | **0.060** |
| **LG29** | **-0.125** | **0.550** | **-0.111** | **0.080** |
| **LG33** | **-0.875** | **0.620** | **-0.167** | **0.120** |
| NCIC1005 | -0.750 | 0.290 | -0.075 | 0.090 |
| **NCIC1009** | **-1.500** | **0.970** | **0.135** | **0.040** |
| *NCIC1012* | *1.125* | *0.167* | *-0.053* | *-0.010* |
| **NCIC2035** | **-0.500** | **0.370** | **-0.335** | **0.120** |
| **SIC015** | **-0.250** | **0.210** | **0.22** | **0.010** |
| **SM24** | **-1.625** | **0.930** | **-0.038** | **0.070** |
| SP022 | -0.375 | 0.140 | -0.414 | 1.450 |
| SP040 | -0.750 | 0.350 | -0.115 | 0.020 |
| **SP051** | **-1.500** | **0.720** | **0.090** | **.** |
| **STJ01** | **0.000** | **0.190** | **-0.246** | **0.000** |
| **STL10** | **0.250** | **-0.050** | **-0.006** | **0.120** |
| **STL18** | **-0.375** | **0.340** | **-0.130** | **0.160** |
| **STL22** | **-1.500** | **0.770** | **-0.013** | **0.150** |
| **STL37** | **0.250** | **0.180** | **0.069** | **-0.010** |
