# Supplementary material for: Six Year Refractive Change among White Children and Young Adults: Evidence for Significant Increase in Myopia among White UK Children
Source: PLoS One. 2016 Jan 19;11(1):e0146332. doi: 10.1371/journal.pone.0146332 (PMC4718680; doi:10.1371/journal.pone.0146332)
Supplement: S3 Table — Outlined below are the Spearman correlations between the change in SER and change in AL, corneal power and ACD. Change in SER vs Change in AL, Spearman’s Correlation, ρ = -0.707, p<0.001. Change in SER vs Change in Corneal Power, Spearman’s Correlation, ρ = -0.114, p = 0.457. Change SER vs Change in ACD, Spearman’s Correlation, ρ = -0.520, p<0.001. (PDF) [file pone.0146332.s003.pdf]

S3 Table

id	Change in SER (DS)	Change in AL (mm)	Change in Corneal Power (D)	Change in ACD (mm)
BB02	0.375	0.280	0.006	-0.060
BB05	-0.250	0.700	-0.284	0.230
BB06	-0.125	0.460	-0.056	0.150
BB17	0.500	0.450	-0.175	0.050
BB25	-0.375	0.800	-0.219	0.170
BB32	-0.625	1.140	-0.252	0.190
BB38	0.625	0.490	0.08	0.090
BKY02	0.000	0.590	-0.128	0.140
BKY05	-0.500	0.760	0.071	0.110
BM08	-1.750	1.030	0.027	0.240
BM19	-1.875	0.800	0.256	0.250
BS01	-0.875	0.410	-0.139	0.030
BS06	1.125	0.200	-0.112	0.090
CH01	-0.875	1.120	0.105	0.170
CH04	-0.375	0.650	-0.003	0.060
CH06	0.750	0.370	-0.587	0.030
CH11	-1.125	1.330	-0.206	0.400
DH14	-1.000	0.750	0.237	0.120
DH18	0.500	0.610	-0.350	0.020
DHCM2006	0.375	0.442	0.071	0.020
DR05	-0.875	0.740	-0.166	0.110
DR07	-1.500	1.120	-0.123	0.180
DR12	-0.750	0.880	-0.230	0.000
DR15	-0.500	1.230	-0.202	0.270
DR17	-0.750	0.800	0.006	0.200
DR22	-0.875	0.800	.	.
GH03	-1.250	1.020	-0.473	0.120
GH04	-0.750	0.560	0.193	0.080
LCEN012	-1.625	0.830	-0.197	0.180
LCEN014	-0.875	0.960	-0.228	0.100
LCEN016	-0.750	0.640	0.127	0.060
LCEN025	0.375	0.630	-0.095	0.160
LCEN027	0.375	0.270	-0.254	-0.030
LIS03	-1.125	0.870	-0.495	0.420
LIS04	-0.375	0.540	-0.372	0.170
LIS14	-0.25	0.820	-0.13	0.180
LIS20	-1.375	0.810	-0.106	0.190
LIS22	-0.25	0.660	-0.267	0.130
LIS24	-0.375	0.820	0.230	0.140
LIS48	-1.375	1.040	-0.067	0.210
PR01	-0.375	0.400	-0.058	0.090
PR02	-1.000	0.580	0.088	-0.090
PR07	0.875	0.520	-0.003	0.080
PR11	-1.250	1.070	-0.359	0.180
PR13	-1.750	1.000	0.016	0.240
PR16	-0.375	0.700	0.105	0.230
