# Supplementary material for: Six Year Refractive Change among White Children and Young Adults: Evidence for Significant Increase in Myopia among White UK Children
Source: PLoS One. 2016 Jan 19;11(1):e0146332. doi: 10.1371/journal.pone.0146332 (PMC4718680; doi:10.1371/journal.pone.0146332)
Supplement: S4 Table — Outlined below are the Spearman correlations between the change in SER and change in AL, corneal power and ACD. Change in SER vs Change in AL, Spearman’s Correlation, ρ = -0.594, p<0.001. Change in SER vs Change in Corneal Power, Spearman’s Correlation, ρ = 0.008, p = 0.964. Change SER vs Change in ACD, Spearman’s Correlation, ρ = -0.322, p = 0.063. (PDF) [file pone.0146332.s004.pdf]

## S4 Table

| id | Change in SER (DS) | Change in AL (mm) | Change in Corneal Power (D) | Change in ACD (mm) |
|---|---|---|---|---|
| BA28 | -0.500 | 0.250 | -0.319 | 0.050 |
| BA65 | -1.375 | 0.230 | -0.028 | -0.200 |
| BC17 | -0.875 | 0.310 | -0.179 | 0.000 |
| CHS12 | -0.250 | 0.290 | -0.615 | 0.030 |
| CI13 | 0.375 | 0.030 | 0.041 | 0.000 |
| CI27 | -0.250 | 0.320 | -0.145 | 0.100 |
| CI58 | 1.125 | -0.020 | -0.139 | -0.040 |
| CI60 | 0.750 | 0.180 | -0.378 | 0.110 |
| ICC17 | -0.250 | 0.210 | 0.032 | 0.020 |
| LC13 | -0.250 | 0.200 | -0.119 | 0.000 |
| LC30 | 0.250 | 0.090 | 0.158 | 0.040 |
| LC34 | 0.375 | 0.270 | 0.134 | -0.020 |
| LHS26 | 0.250 | 0.130 | -0.169 | -0.040 |
| NCIC1013 | 1.250 | -0.144 | 0.055 | -0.040 |
| NCIC2034 | 0.000 | 0.300 | -0.255 | 0.060 |
| NCIC2040 | -1.000 | 0.230 | -0.406 | 0.010 |
| O01 | 1.000 | 0.110 | -0.222 | -0.160 |
| O07 | -0.250 | 0.200 | 0.108 | -0.070 |
| SIC034 | 0.125 | 0.230 | -0.163 | -0.050 |
| SIC066 | 1.000 | 0.250 | 0.059 | 0.060 |
| SIC073 | 1.750 | -0.260 | -0.082 | -0.080 |
| SM26 | 0.875 | 0.250 | -0.115 | -0.050 |
| SM27 | -0.125 | 0.310 | -0.049 | -0.040 |
| SM30 | -1.250 | 0.730 | -0.086 | 0.040 |
| SP007 | -0.125 | -0.030 | -0.054 | 0.0900 |
| SP014 | 0.625 | 0.036 | -0.583 | -0.110 |
| SP017 | -0.375 | 0.266 | 0.383 | 0.020 |
| SP025 | 0.500 | 0.210 | -0.278 | 0.000 |
| SP031 | 0.125 | 0.180 | 0.123 | 0.060 |
| SP042 | -0.250 | 0.234 | 0.096 | 0.000 |
| SP060 | -1.625 | 0.210 | 0.045 | 0.050 |
| SP066 | 0.125 | 0.094 | 0.026 | -0.070 |
| STL11 | 0.750 | 0.130 | -0.283 | 0.000 |
| STL34 | 0.875 | -0.090 | 0.130 | -0.110 |
